# Supplementary material for: High-resolution structure determination of sub-100 kDa complexes using conventional cryo-EM
Source: Nat Commun. 2019 Mar 4;10:1032. doi: 10.1038/s41467-019-08991-8 (PMC6399227; doi:10.1038/s41467-019-08991-8)
Supplement: Supplementary file 1 — Supplementary Information [file 41467_2019_8991_MOESM1_ESM.docx]

**Supplementary Note**

A notable commonality that has emerged from the high resolution structure determination of many different types of samples by the cryo-EM community is that relatively few (e.g. <20%) of the initially picked “particles” are retained in the final high resolution reconstruction(s). This trend seems to be particularly true for the smaller macromolecules presented here, with only ~2% of the ADH or ~7% of metHb particles selected from 2D classification contributing to the final reconstructions. This is a considerable loss of potentially useful structural information, but it may also alternatively suggest that only a subset of collected micrographs contain the “ideal” high-resolution particles, i.e. those that are minimally impacted by radiation damage or beam-induced motion. To address this latter point, we investigated whether the particles comprising the final reconstructions of ADH and metHb were equally distributed across all micrographs or obtained from a small subset of images.

For ADH, 659,662 particles picked from 1,151 micrographs were retained after 2D classification (~570 particles/micrograph). Subsequent rounds of refinement and 3D classification yielded a smaller particle stack (80,060 particles) distributed across 1138 micrographs (~70 particles/micrograph), demonstrating that while substantially fewer particles were retained, almost all micrographs were represented in the dataset. This particle stack produced a ~3 Å resolution reconstruction, and an additional round of 3D classification and local CTF refinement identified 11,672 particles that yielded a ~2.7 Å resolution reconstruction. This final subset of particles represents less than 2% of the initially selected data but originates from 564 unique micrographs, thus indicating that the highest resolution particles are spread across a large subset of images. Importantly, all micrographs contributing to the final stack of particles originated from all 23 squares that were imaged to generate the 1,151-micrograph dataset.

The same trend was observed for the metHb dataset. A total of 513,632 metHb particles extracted from 1,665 micrographs (~308 particles/micrograph) were retained from the first round of 2D classification. An additional round of 2D classification yielded 160,169 particles originating from all 1,665 micrographs (~96 particles/micrographs) that refined to ~3.0 Å resolution. Subsequent rounds of 3D classification and auto-refinement yielded two reconstructions at ~2.8 Å and ~3.2 Å resolution. Together, these structures comprise 35,809 particles originating from 1362 micrographs (~26 particles/micrograph). Importantly, all the micrographs in the final metHb structures originated from the 27 squares that contained particles after 2D classification.

For both ADH and metHb, the number of squares comprising useable micrographs remains unchanged throughout data processing but the micrographs per square ratio and the number of “useable” particles per micrograph decrease substantially as processing proceeds. A simple explanation for this observation is that the micrographs eliminated during 2D and 3D classification contained substandard ice; e.g. ice that that was either too thick, resulting in particles with poor signal-to-noise ratio, or slightly too thin, thus damaging the high-resolution features of the particles. An important observation from these analyses is that few particles from many micrographs contribute to the final reconstructions, rather than a large number of particles from a few micrographs. This suggests that the particles included in the final reconstructions are not only the most conformationally homogenous, but also the least impacted by factors affecting high-resolution information (e.g. improper vitrification, destabilizing or denaturing interactions at the air-water interface, ice thickness, beam-induced motion and/or radiation damage; etc.), and therefore have the most accurate CTF and noise estimates.

We thus speculate that various factors (e.g. false picks, damaged/denatured particles, poor signal-to-noise ratio) affect all stages of refinement but vary in their contributions depending on how far data processing has progressed. Specifically, we speculate that particle elimination during earlier stages of processing (e.g. binned 2D classification) is dominated by the removal of false positives and particles with gross errors in CTF correction and/or noise estimates. Later-stage processing steps for both ADH and metHb datasets to obtain the “highest-resolution” particles further eliminated a majority of the remaining particles (78-85%) with only a modest gain in nominal map resolution (e.g. ~0.3 Å resolution). This leads us to believe that only the most conformationally homogenous particles with proper noise estimates and minor, if any, errors in CTF parameters comprise the highest-resolution reconstructions. We further speculate that these “high-resolution” particles are primarily responsible for the observed resolution estimate at intermediate stages of processing and moreover strongly influence the overall b-factor of the data (Supplementary Fig. 7). Indeed, 3D auto-refinement of the ADH particles that were deselected after the final round of 3D classification (~68K particles) resolved to ~9.2 Å whereas the input particle stack (~80K particles) refined to ~3 Å resolution.

Advances in metadata analysis that enable more detailed monitoring of particles at each stage of processing will aid in better understanding the factors contributing to low particle retention in final cryo-EM reconstructions. Further, more powerful classification approaches or imaging technologies will help elucidate whether the vast majority of particles that are removed during processing in fact represent molecules in a wide range of slightly different conformational states from the final structure.

|  | **Alcohol Dehydrogenase** | **Methemoglobin** |
| --- | --- | --- |
|  |  |  |
| **Post-2D classification** |  |  |
| Particles | 659,662 | 513,632 |
| Particles/Micrograph | 573 | 308 |
| Micrographs | 1,151 | 1,665 |
| Micrographs/Square | 50 | 61 |
| Squares | 23 | 27 |
|  |  |  |
| **Intermediate Processing** |  |  |
| Particles | 80,060 | 160,169 |
| Particles/Micrograph | 70 | 96 |
| Micrographs | 1,138 | 1,665 |
| Micrographs/Square | 49 | 61 |
| Squares | 23 | 27 |
|  |  |  |
| **Final Structure(s)** |  |  |
| Particles | 11,672 | 35,809 |
| Particles/Micrograph | 20 | 26 |
| Micrographs | 564 | 1362 |
| Micrographs/Square | 24 | 50 |
| Squares | 23 | 27 |

**Supplementary Figures**


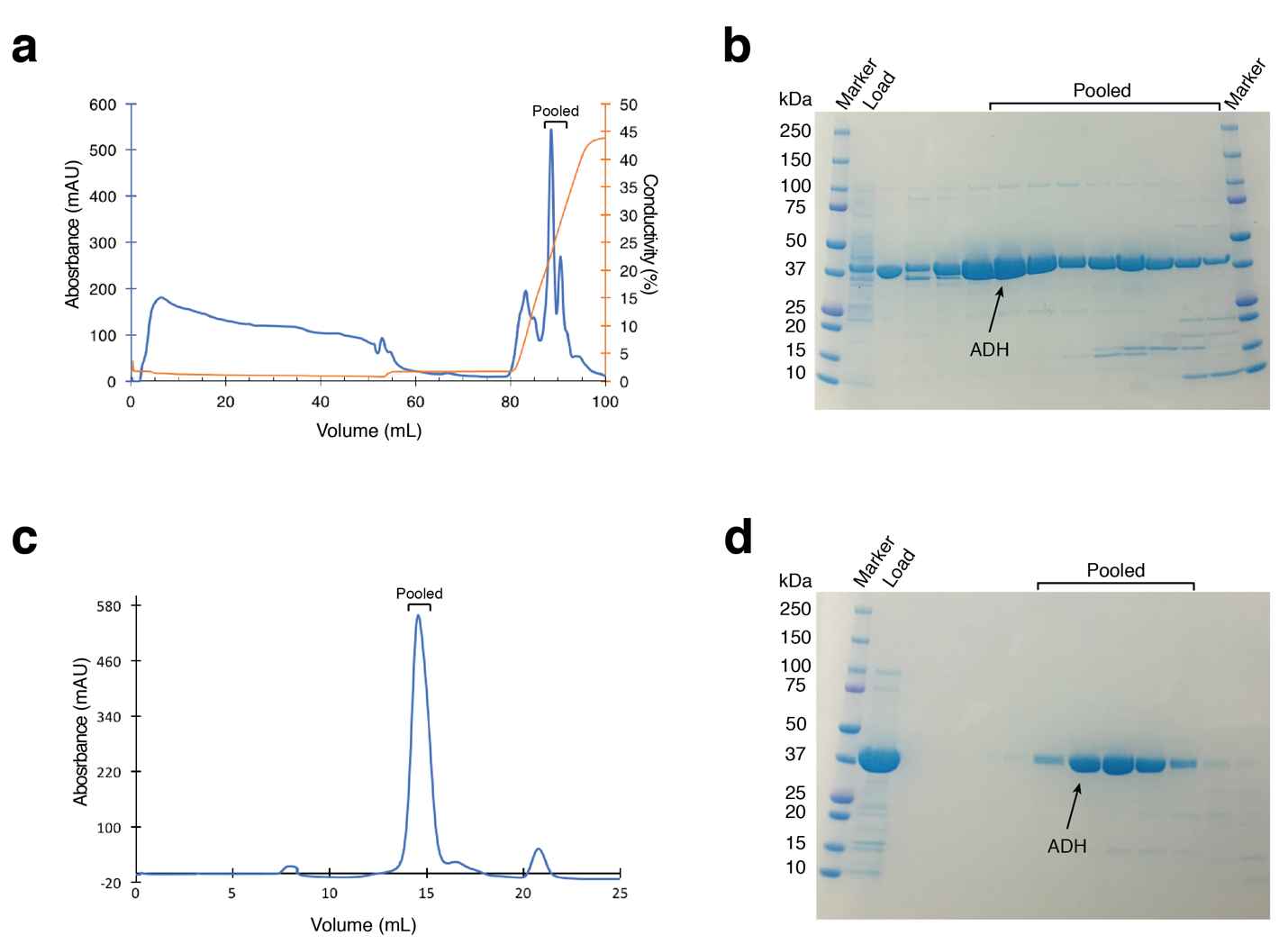


**Supplementary Figure 1. Purification of alcohol dehydrogenase. (a)** Elution profile of alcohol dehydrogenase (ADH) from a HiTrap SP sepharose cation exchange column. Traces for UV absorbance (mAU) (blue) and conductivity (%) (orange) are shown. Fractions that were pooled are indicated. **(b)** SDS-PAGE analysis of ADH elution from HiTrap SP sepharose shown in (a). Molecular weight marker, load, and pooled fractions are indicated. **(c)** Elution profile of ADH from a Superdex 200 gel filtration column. UV absorbance (mAU) is shown as a blue trace. Fractions that were pooled are indicated. **(d)** SDS-PAGE analysis of the gel filtration run shown in (c). Molecular weight marker, load, and pooled fractions are indicated.


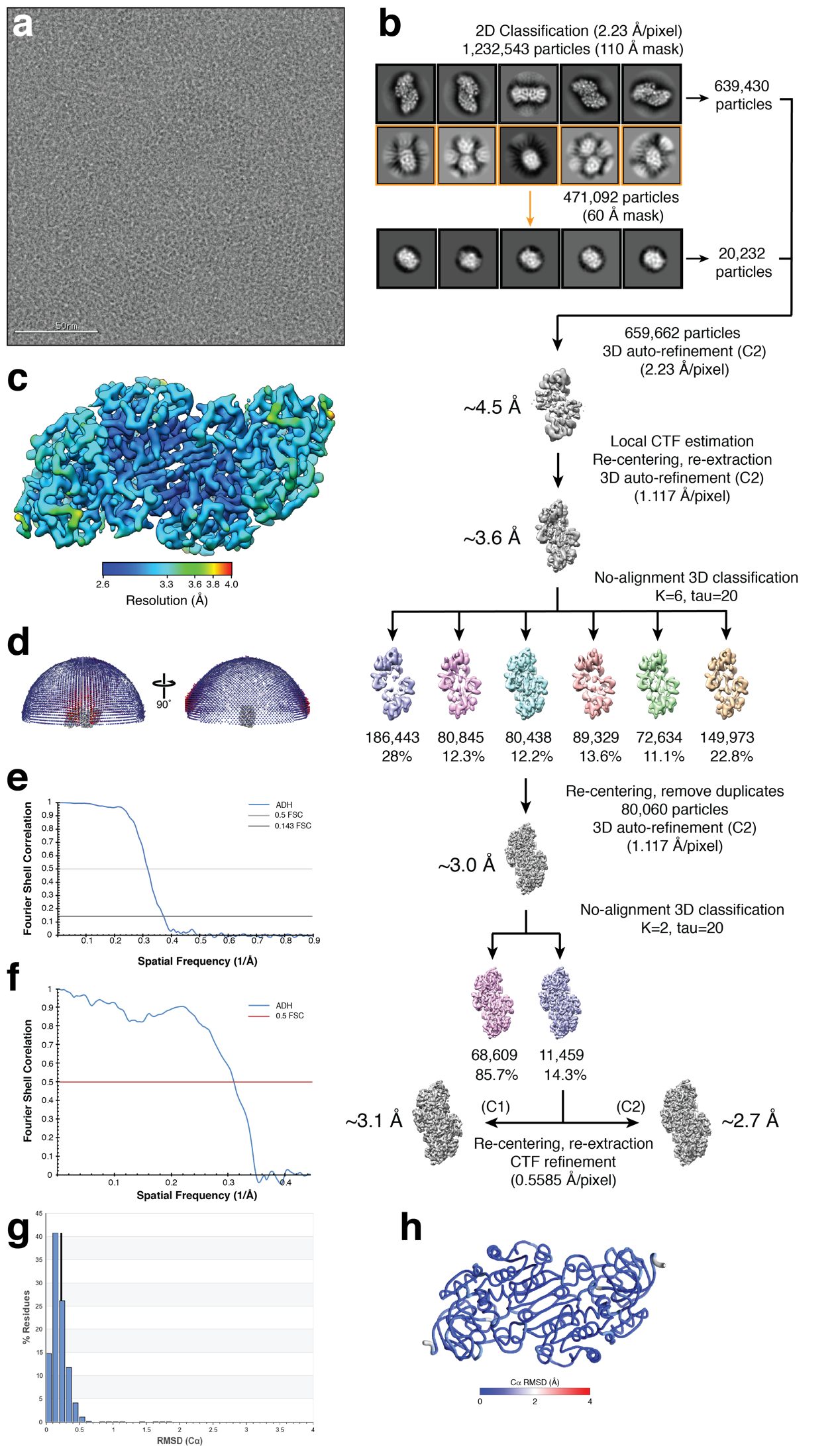


**Supplementary Figure 2. Schematic for alcohol dehydrogenase single-particle cryo-EM data processing. (a)** Representative motion-corrected micrograph of vitrified ADH collected at ~1 µm underfocus. **(b)** ~1.2 million particles were extracted from the aligned, dose-weighted micrographs, Fourier binned 4 x 4, and subjected to two subsequent rounds of reference-free 2D classification using RELION^1^. 2D classes highlighted in yellow were selected for a second round of 2D classification using a smaller soft circular mask (60 Å vs. 110 Å) to discern orthogonal views of ADH. Representative 2D class averages are shown. Particles comprising the “best” classes were 3D auto-refined to ~4.5 Å resolution followed by local CTF estimation, particle coordinate re-centering, and re-extraction Fourier binned 2 x 2. 3D auto-refinement of these particles yielded a ~3.6 Å resolution reconstruction that was then subjected to no-alignment 3D classification (tau_fudge=20) and the best resolved class (80,060 particles) was further auto-refined to ~3.0 Å resolution. Following another no-alignment 3D classification, particles (11,451) composing the “best” resolved class were re-centered and re-extracted unbinned. Refinement of per-particle defocus and beam tilt yielded a final reconstruction at ~2.7 Å resolution (C2 symmetry) or ~3.1 Å resolution (C1 symmetry). **(c)** ADH EM density colored by local resolution (estimated using BSOFT^2^). **(d)** Plot showing the Euler angle distribution of the final ADH EM density. **(e)** Gold-standard^3-5^ Fourier shell correlation (FSC) curve generated from the independent half maps contributing to the ~2.7 Å resolution ADH EM density. **(f)** FSC curve calculated between the ADH EM density and the refined atomic model. **(g)** Histogram of the per-residue Cα RMSD values calculated from the top 10 refined ADH atomic models. **(h)** Worm plot representation of the per-residues Cα RMSD calculated from the top 10 refined ADH atomic models.


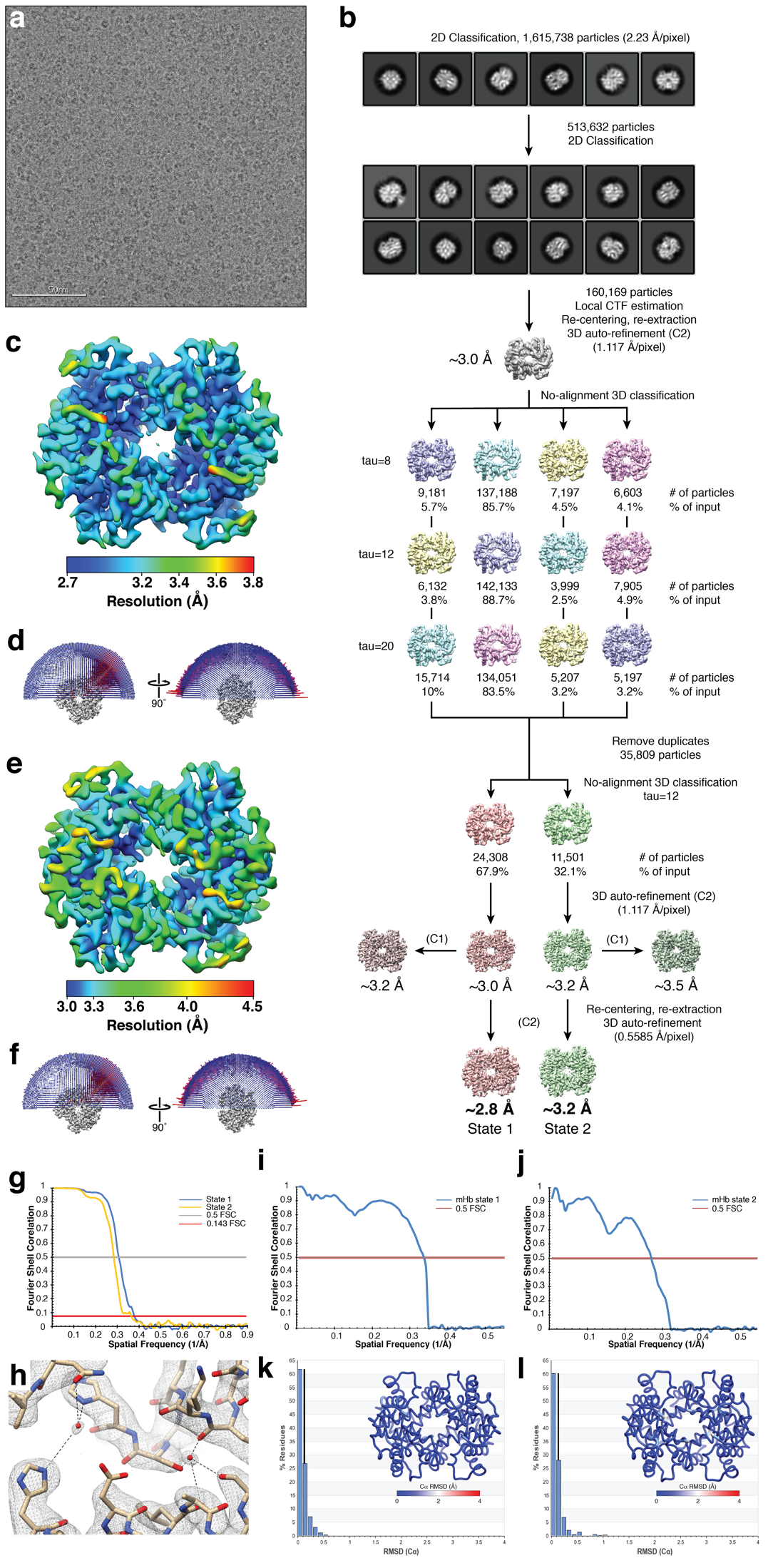


**Supplementary Figure 3. Schematic for methemoglobin single-particle cryo-EM data processing.** **(a)** Representative motion-corrected micrograph of vitrified methemoglobin (mHb) collected at ~1 µm underfocus. **(b)** ~1.6 million particles were extracted from the aligned, dose-weighted micrographs, Fourier binned 4 x 4, and subjected to two subsequent rounds of reference-free 2D classification using RELION^1^. Representative 2D class averages are shown. Particles comprising the “best” classes (160,169) were used for local CTF estimation, particle coordinate re-centering, and re-extraction Fourier binned 2 x 2. 3D auto-refinement of these particles yielded a ~3.0 Å resolution reconstruction that was then subjected to three parallel no-alignment 3D classifications (tau_fudge values of 8, 12, or 20). Unique particles corresponding to the best resolved classes from each classification were combined (35,809 particles) and further 3D auto-refined to ~3.0 Å resolution. Another no-alignment 3D classification of these particles yielded two conformationally distinct classes (state 1 and state 2) that 3D auto-refined to ~2.8 and ~3.2 Å resolution, respectively, with C2 symmetry applied. **(c)** and **(e)** mHb state 1 and state 2 EM densities, respectively, colored by local resolution (estimated using BSOFT^2^). **(d)** and **(f)** Plots showing the Euler angle distribution for the final mHb state 1 and state 2 EM densities, respectively. **(g)** Gold-standard FSC^3-5^ curves generated from the independent half maps contributing to the ~2.8 Å (state 1, blue trace) or the ~3.2 Å resolution (state 2, orange trace) mHb EM densities **(h)** Ordered water molecules (red spheres) in the EM density of mHb state 1. Putative hydrogen bonds are shown as black dotted lines. **(i)** FSC curve calculated between the mHb state1 EM density and the refined atomic model. **(j)** FSC curve calculated between the mHb state 2 EM density and the refined atomic model. **(k)** Histogram of the per-residue Cα RMSD values calculated from the mHb state 1 top 10 refined atomic models. Inset is a worm plot representation of per-residue Cα RMSD calculated from the top 10 refined mHb state 1 atomic models. **(l)** Histogram of the per-residue Cα RMSD values calculated from the mHb state 2 top 10 refined atomic models. A worm plot representation of per-residue Cα RMSD calculated from the top 10 refined mHb state 2 atomic models is shown (inset).

**
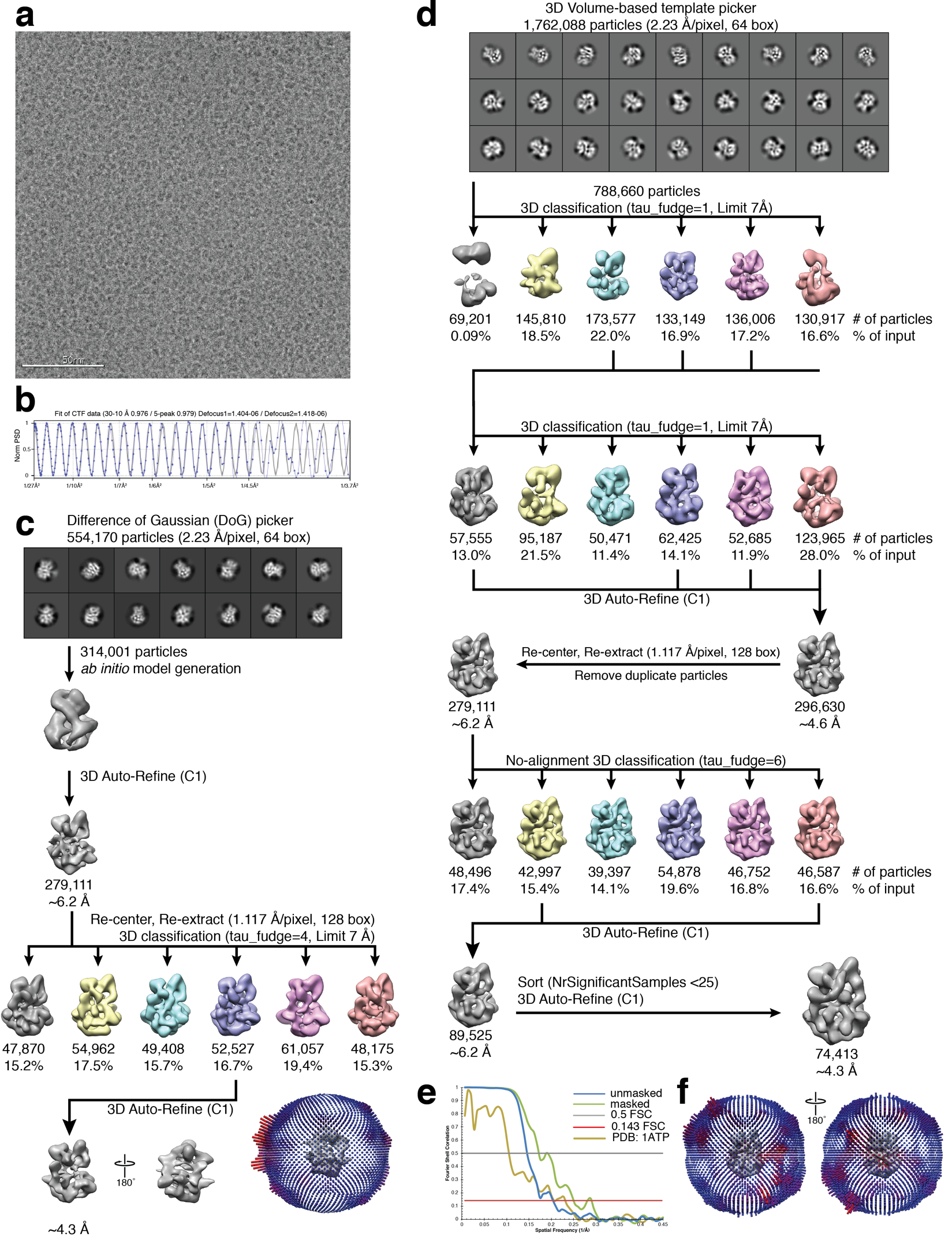
**

**Supplementary Figure 4. Schematic for protein kinase A catalytic domain-IP20 complex single-particle cryo-EM data processing. (a)** Representative motion-corrected micrograph of vitrified iPKA_c_ tilted at 30˚ and imaged at ~1.4 µm underfocus. **(b)** 1-dimensional plot of the contrast transfer function (CTF) Thon rings (black line) and the CTF estimated with CTFind^6^ (blue line). **(c)** ~554K DoG-picked particles obtained from untilted data collection were extracted from the aligned, dose-weighted micrographs, Fourier binned 4 x 4 (2.234 Å/pixel, 64-pixel box), and subjected to reference-free 2D classification using RELION^1^. Representative 2D class averages are shown. Particles comprising the “best” classes were 3D auto-refined (C1 symmetry) using an *ab initio* model created using cryoSPARC^7^. Particles were re-centered and re-extracted, Fourier binned 2 x 2 (1.117 Å/pixel, 128-pixel box) and subsequently 3D classified (tau_fudge=4, E-step limit=7 Å). The best-resolved class was 3D auto-refined to ~4.3 Å resolution, as estimated by gold-standard FSC^3-5^. Plot showing the Euler angle distribution. **(d)** ~1.76 million particles from the combined untilted and tilted data, picked using a 3D-volume-based template picker in RELION^1^, were extracted from the aligned and dose-weighted micrographs, Fourier binned 4 x 4 (2.234 Å/pixel, 64-pixel box), and subjected to RELION^1^ reference-free 2D classification. Representative 2D class averages are shown. Particles comprising the “best” classes were subjected to two rounds of 3D classification (tau_fudge=1, E-step limit=7 Å). The “best” resolved classes were 3D auto-refined (C1 symmetry) to yield a ~4.6 Å resolution reconstruction, as estimated by gold-standard FSC^3-5^. Particles were re-centered and re-extracted, Fourier binned 2 x 2 (1.117 Å/pixel, 128-pixel box) and duplicate particle picks were eliminated. These particles were 3D auto-refined to ~6.2 Å resolution and subjected to no-alignment 3D classification (tau_fudge=6). The “best” resolved classes were combined and 3D-auto-refined to ~6.2 Å resolution. Particles with NrOfSignificantSamples >25 were eliminated and 3D auto-refined to ~4.3 Å resolution. **(e)** Gold-standard FSC^3-5^ curves (masked and unmasked) generated from the independent half maps contributing to the ~4.3 Å resolution iPKA_c_ EM density and FSC curve between the final iPKA_c_ EM density and rigid-body fit model (PDB ID: 1ATP [http://dx.doi.org/10.2210/pdb1ATP/pdb]). **(f)** Plots showing the Euler angle distribution of the final iPKA_c_ EM density.

**
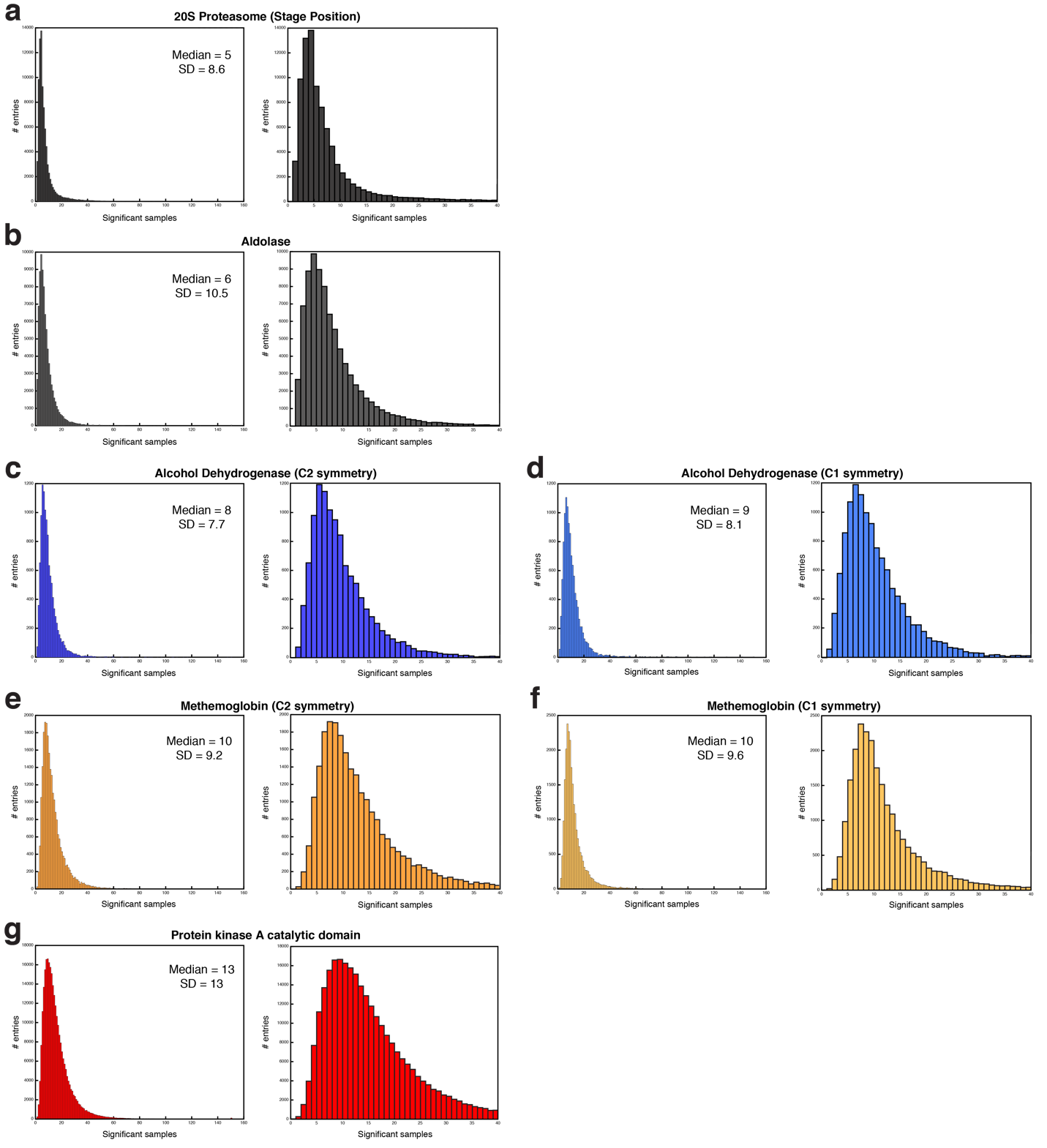
Supplementary Figure 5. Distribution of RELION significant samples.** Histogram plots of the number of significant samples of each particle contributing to the final cryo-EM reconstructions (left, 160 maximum; right, zoomed 40 maximum) of **(a)** 20S proteasome (EMDB ID: 8741), **(b)** aldolase (EMDB ID: 8743), **(c)** ADH C2-symmetric and **(d)** C1-symmetric refinements, **(e)** mHb State 1 C2-symmetric and **(f)** C1-symmetric refinements, and **(g)** iPKA_c_. The median and standard deviation values are reported.


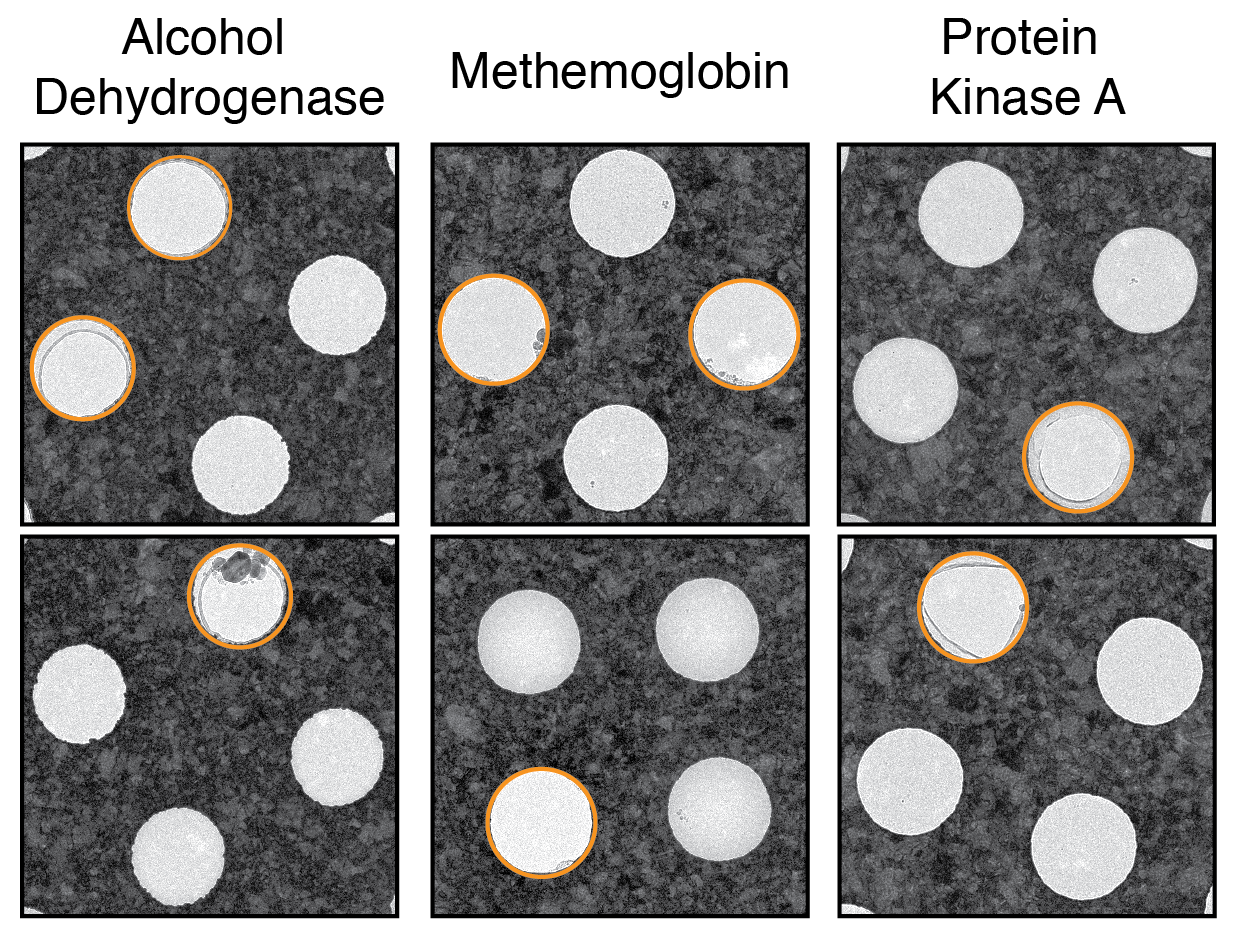


**Supplementary Figure 6. Medium-magnification images used for data collection.** Representative images of ADH, metHb, and PKA specimen grids collected at 1200x magnification depicting the thickness of the vitreous ice that was targeted for exposure. Holes without useable ice are indicated in orange.


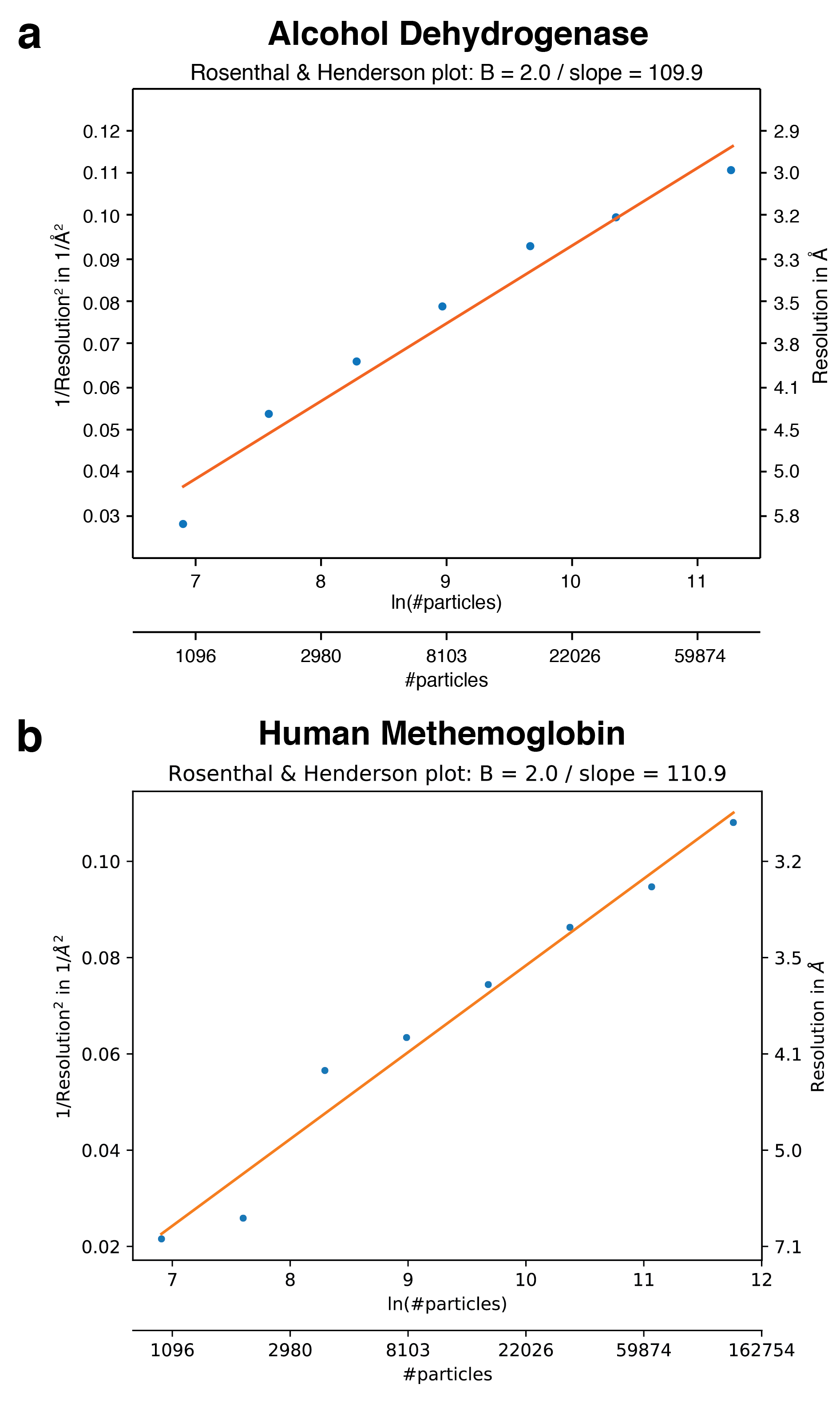


**Supplementary Figure 7. Rosenthal and Henderson B-factor plots.** 3D refinements of subsets of increasing sizes were performed for **(a)** ADH and **(b)** mHb state 1, and B-factor plots^8^ were subsequently generated by plotting the inverse of the squared resolution against the natural logarithm of the number of particles. The B-factor for each data set was estimated by fitting a straight line through the plot points.

**Supplementary Table 1. Cryo-EM data collection, refinement, and validation statistics**

**References:**

1. Scheres, S. H. W. RELION: implementation of a Bayesian approach to cryo-EM structure determination. *J Struct Biol* **180,** 519–530 (2012).

2. Cardone, G., Heymann, J. B., Steven, A. C.Steven. One number does not fit all: Mapping local variations in resolution in cryo-EM reconstructions. *J Struct Biol* **184,** 226–236 (2013).

3. Henderson, R. *et al.* Outcome of the first electron microscopy validation task force meeting. in **20,** 205–214 (2012).

4. Scheres, S. H. W. & Chen, S. Prevention of overfitting in cryo-EM structure determination. *Nat Methods* **9,** 853–854 (2012).

5. Chen, S. *et al.* High-resolution noise substitution to measure overfitting and validate resolution in 3D structure determination by single particle electron cryomicroscopy. *Ultramicroscopy* **135,** 24–35 (2013).

6. Rohou, A. & Grigorieff, N. CTFFIND4: Fast and accurate defocus estimation from electron micrographs. *J Struct Biol* **192,** 216–221 (2015).

7. Punjani, A., Rubinstein, J. L., Fleet, D. J. & Brubaker, M. A. cryoSPARC: algorithms for rapid unsupervised cryo-EM structure determination. *Nat Methods* **14,** 290–296 (2017).

8. Rosenthal, P. B. & Henderson, R. Optimal Determination of Particle Orientation, Absolute Hand, and Contrast Loss in Single-particle Electron Cryomicroscopy. *Journal of Molecular Biology* **333,** 721–745 (2003).

9. Barad, B., Echols, N.A., Wang, R. Y., Cheng, Y., DiMaio, F., Adams, P. D. & Fraser, J. S. EMRinger: side chain-directed model and map validation for 3D cryo-electron microscopy. *Nat Methods* **12**(10): 943-946 (2015).
